# Citizen science and expert opinion working together to understand the impacts of climate change

**DOI:** 10.1371/journal.pone.0273822

**Published:** 2022-08-30

**Authors:** Maria Isabel Garcia-Rojas, Marie R. Keatley, Nadiah Roslan

**Affiliations:** 1 Earthwatch Institute, Melbourne, Victoria, Australia; 2 School of Agricultural, Environmental and Veterinary Sciences, Charles Sturt University, Bathurst, New South Wales, Australia; 3 School of Ecosystem and Forest Sciences, The University of Melbourne, Creswick, Victoria, Australia; 4 Australian Museum Research Institute, Australian Museum, Sydney, New South Wales, Australia; Chinese Academy of Sciences, CHINA

## Abstract

In the absence of historical information on phenology available in Australia, expert opinion was used for selecting indicator species that would be suitable for monitoring phenology on a continental scale as part of ClimateWatch—a citizen science program. *Jacaranda mimosifolia* being the most frequently observed species was used in this study to test expert opinion and the adequacy of citizen science records in detecting the influence of climatic conditions on this species’ flowering phenology. Generalised Additive Models for Location Scale and Shape were used to explore the occurrence and intensity of flowering of Jacaranda in relation to rainfall, temperature, and sun exposure. Jacaranda flowering onset was influenced by winter cold exposure, while flowering intensity was related to increasing sun exposure as spring progresses, and both were influenced by the conditions for flowering in the former flowering seasons (i.e., sun exposure and highest temperatures reached, respectively). Our models provide the first attempt to describe the climate drivers for *Jacaranda mimosifolia* flowering in the southern hemisphere and identify where climatic changes will most likely alter this tree’s phenology in Australia and benefit or challenge its reproductive ability. They also support the choice of species for citizen science programs based on expert opinion.

## Introduction

Phenology is the study of recurrent biological events in nature and how these relate to seasonal and interannual variations in climate [1]. In plants this can include events such as flowering and fruiting, and in animals this can include reproduction, hibernation, or migration. In plants, it is well understood that the timing of phenology is primarily driven by climate (largely temperature and rainfall) [2]. As our climate is changing with increasing temperature, altered rainfall regimes, and heightened frequency of extreme events, many phenological events are occurring earlier [3–5].

The effect of climate change on phenology has implications for the natural world. Species interactions, ecosystem functioning, and individual species survival can all be impacted [5–8]. Mismatches in the phenological events of co-dependent species such as predators and prey, pollinators and plants, can cause disruption in food webs and the supply of food and natural resources [9, 10]. Climate change impacts on phenology can also have implications for human well-being and culture. Warming temperatures can change the timing of flowering and the amount of pollen in the air, altering allergenic pollen season [11, 12]. Phenological shifts can also impact agricultural production and the timing of flower festivals which attract high numbers of tourists. As such, changes in the timing of phenological events are important to monitor to detect detrimental effects of climate change on human and natural systems.

A powerful method for monitoring and recording phenological changes over large spatial and temporal scales involves public participation in observing nature through citizen science [13, 14]. The main body of impactful research from citizen science phenological programs to date, however, are largely restricted to the northern hemisphere [3, 4, 15]. In response to the lack of phenological records in Australia, in 2009, Earthwatch Australia together with the Bureau of Meteorology and University of Melbourne, initiated a citizen science network called ClimateWatch. Like other regions of the world, common phenological datasets in Australia are those that have recorded flowering plants, migrating birds, as well as crop species such as wine-grapes [5]. The Australian public has been collecting phenological and species presence data through the ClimateWatch project for the past 10 years. Today it stands as one of the few continental-scale citizen science phenology networks in the southern hemisphere.

The governance structure of ClimateWatch includes a Science Advisory Panel (SAP) comprising of senior climate change biologists in Australia. The SAP advised on the selection of over 170 taxa to establish an indicator species list for ClimateWatch. Species for citizen science phenological programs are chosen to meet a set of criteria–common among them are: safe to observe, easy to identify and unlikely to be confused with another species, easily recognisable phenophases, sensitivity to climate typically air temperature and a broad geographic range [16, 17]. Using these criteria, the selected species for ClimateWatch were mostly native to Australia. However, non-native species were also included as most of Australia’s population is urban [18] with gardens and street trees dominated by non-native species [19, 20]. These species were also selected to have overlap with species being measured or considered by phenology networks in other countries. Jacaranda (*Jacaranda mimosifolia*) an iconic, non-native street-tree in Australia and across the globe, was selected for ClimateWatch for these reasons and is the focal species of this paper. For the purpose of this study, Jacaranda will refer to *Jacaranda mimosifolia* unless stated otherwise.

Jacaranda is a monsoonal, deciduous tree from South America (i.e. Argentina, Bolivia, Brazil, Paraguay and Uruguay) [21]. It has been introduced to over 80 countries (e.g. Australia, Greece, India, Kenya, Lebanon, South Africa, Spain, Portugal, Uganda, United States, Tanzania) primarily as an ornamental tree because of its distinctive blue-purple flowers that start to flower from Spring through to Summer [22–24]. It is believed to have been planted in Australia in the 1850s [25]. Under the set criteria stated above, the ClimateWatch SAP assumed that its distinctive blue-purple flowers supported ease of identification and reduced potential for observational error. Moreover, its wide ornamental planting across Australia was assumed to result in high likelihood of detection and monitoring by the community. Its use as a tree that citizen scientists could easily monitor flowering status provided an opportunity for comparison of the impact of climate change at a large scale across Australian cities and other parts of the world. However, the hypothesis that its flowering phenology is influenced by climate has been seldom examined [e.g., 23]. This paper explores this question, in particular: can the influence of climate on phenology be derived from Australian citizen science records? Is expert opinion effective in the selection of citizen science indicator species? What are the climate drivers for Jacaranda in Australia?

## Materials and methods

### Data collection and validation

Observational ClimateWatch data [26] were collected on a casual basis or along designated ClimateWatch trails across Australia. Observations were submitted to the ClimateWatch database by registered users through the free ClimateWatch mobile app or website (www.climatewatch.org.au). No private information was obtained, and records were de-identified in line with the ClimateWatch Privacy Policy (https://www.climatewatch.org.au/privacy-policy). Information on the indicator species, including images and identification field guides were made available to ClimateWatch users through the app and website. Observations were entered through a series of drop-down menus and text fields. Overall, citizen scientists collected 11,578 Jacaranda observations across Australia between October 2009 and December 2019.

**Fig 1:**
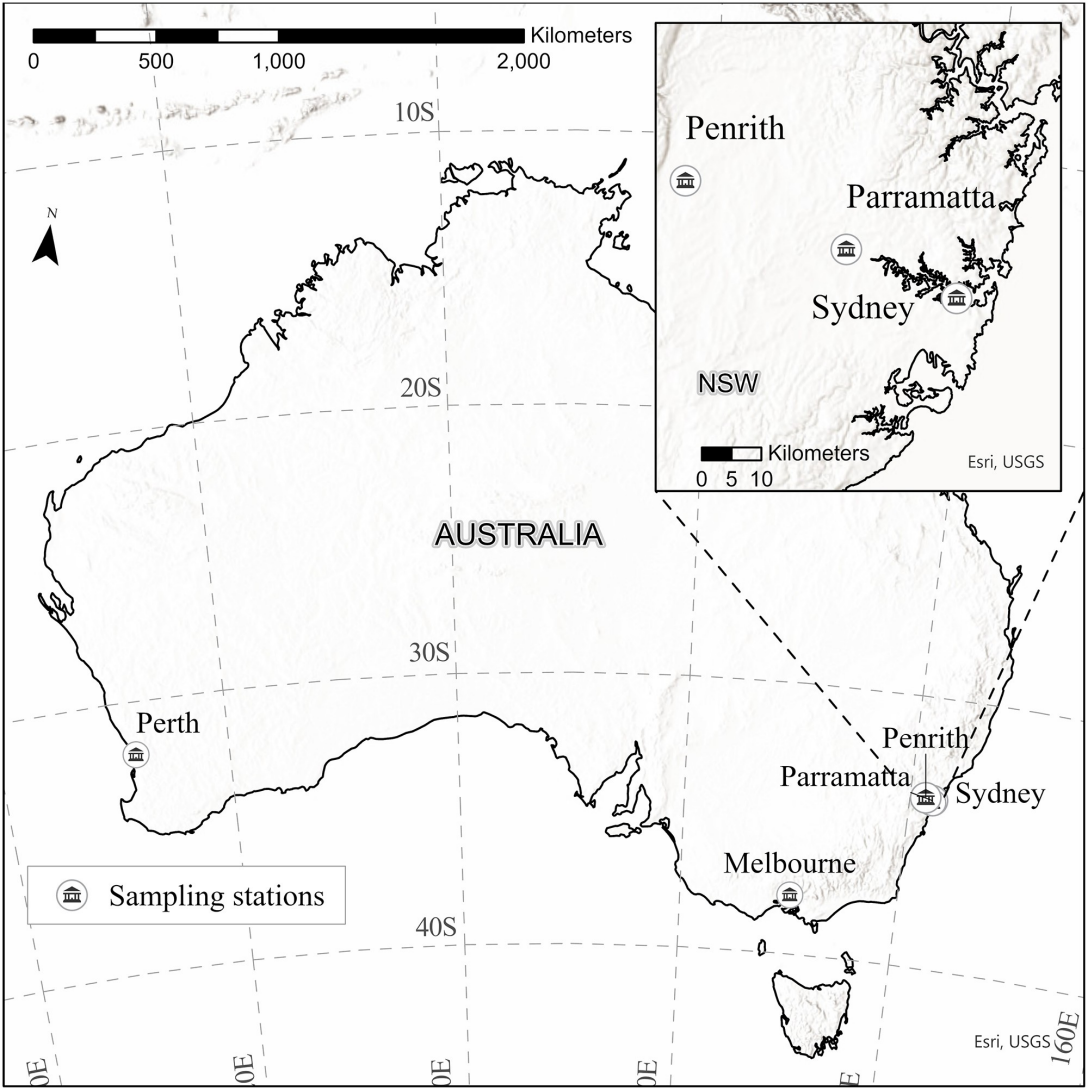
Weather sampling stations chosen to explore Jacaranda relationships to climate signatures between 2009–2019 in Australia. (a) Location of all stations at a country level. (b) Location of stations within New South Wales State. Image created by the authors in ESRI’s ArcGIS Pro using basemaps supported by Esri under a CC BY license, original copyright 1995–2022 Esri.

Five locations where Jacaranda observations were most concentrated were used in this study to explore the underlying seasonal flowering patterns of this species and the relationship to climate. Weather stations within 15km were initially assessed, and subsequently reduced to 7.5km as closer weather stations became apparent. Jacaranda observations located within 7.5km of the following weather stations were assigned to that station and grouped together: Melbourne Regional Office, Perth Regional Office, Sydney Observatory, Parramatta North and Penrith Lakes (hereafter referred to as Melbourne, Perth, Sydney, Parramatta and Penrith, respectively) (Fig 1).

Jacaranda observations with associated images were validated by trained volunteers. Only validated records located within 7.5km of a weather station which contained phenological information (flowering status) (i.e. 3,292 records accounting for 29% of the overall Jacaranda observations) were used in these analyses.

### Flowering period and intensity

Potential flowering period were explored for each station across the sampling period (2009–2019), by plotting the monthly occurrence of flowering and non-flowering. Records including flowering observations for each station, month and year were used to calculate flowering intensity (ranging from 0 to 1), as the proportion of observations where there was flowering detected, over those where there were non-flowering trees detected. A score of 0 indicated that no flowering occurred whilst a score of 1 indicated that flowering was dominant and flowers were seen in all trees at that location, during the particular month and year. Records where flowering information was not given by citizen scientists were excluded when calculating flowering intensity.

Measuring flowering intensity provided a means to standardise data used in analyses, and manage differences in observation frequency related: to citizen science events unevenly covering the 10 years of sampling effort, weather conditions deterring data collection, citizen science variable commitment to the program, and different population density at sampling stations. Measuring flowering intensity as a proportion reduced potential noise and ensured differences in sampling effort did not have a high influence on the analysis’ results.

### Local climate conditions

First, monthly climatic averages were used to model the relationships between flowering of Jacaranda and climate. Rainfall, mean minimum temperature, mean maximum temperature, lowest temperature, highest temperature, and solar exposure were extracted by month from January 2009 to December 2019 from the Australian Government Bureau of Meteorology for each of the five weather stations chosen: Melbourne, Perth, Sydney, Parramatta and Penrith (Fig 1). Monthly measures of the Southern Oscillation Index (SOI) were extracted from 2009–2019 from the Australian Government Bureau of Meteorology [27] to account for potential influences of the intensity of El Niño or La Niña events in the Pacific Ocean on flowering of Jacaranda trees. In eastern Australia, positive SOI values are indicative of La Niña cooler conditions and increased chances of flood, whilst negative values refer to El Niño warmer and drier conditions.

Due to the closure of the Bureau of Meteorology’s Melbourne Regional Office in 2015, data from the Royal Botanical Gardens and Olympic Park (nearby stations within 10km from Melbourne Regional Station) were used to fill the gaps in climate data and compile a single time-series. In doing so, local weather trends and their potential relationship with Jacaranda flowering could be explored between 2009 and 2019. Where climate data were missing for a particular month (i.e., less than 5% of the data), a value was extrapolated by calculating the average between the previous and following month, during which the data gap was located.

Secondly, general climate trends were described using long-term averages on rainfall, temperature and solar exposure (30-years) for all five weather stations, to provide a basis from which to assess current local climate patterns and discuss potential future shifts resulting from changes in local climates. These long-term trends were obtained from average maps calculated by the Australian Government Bureau of Meteorology for the entire country from thousands of weather stations [28–30]. Descriptive ranges, means and trends were extracted for the five stations using ArcGIS Pro. Temperature and rainfall averages were calculated by the Australian Government Bureau of Meteorology for the 30-year period between 1961–1990 (which is the most recent standard reference period identified by the World Meteorological Organization) [31]. Average solar exposure was only available for the period between 1990 to 2019 (i.e., once satellites were launched) [31].

### Flowering responses to climate

The GAMLSS framework of statistical modelling (gamlss.nl package) accessed from the R library [32] was used with a cubic spline smoothing function and a forwards-stepwise stepGAIC function in GAMLSS [33] to explore the relationship between the seven climatic variables (including rainfall, mean minimum temperature, mean maximum temperature, lowest temperature, highest temperature, solar exposure, and the Southern Oscillation Index) and flowering of Jacaranda trees across stations. Station was used as an additional variable to detect potential differences in flowering relating to location. Lagged dependencies of flowering with prior climatic events (up to 12 months prior) were extracted for each month, added to the data frame, and included in the stepwise stepGAIC function.

Two types of models were run to explore the relationships between Jacaranda flowering and climate. The first one used a binomial distribution and classified each month (per year per station) as either flowering or non-flowering, assigning flowering to a month even when there was only one tree observed with flowers, and non-flowering only to those months where no flowers were observed at all. The second type of models tested relationships between flowering intensity and climatic variables to determine the mixture of conditions most favourable for Jacaranda flowering. Flowering intensity was fitted to 13 different distribution families and used the lowest AIC and lower degrees of freedom to determine the best fit and choose the most appropriate distribution to run these models. Tests show that the Beta distribution (Table 1) and the algorithm the RS [34] were best suited to explore trends in flowering intensity. These models included only months and stations where there had been at least one flowering observation. Hierarchical partitioning accessed from the R library [32] was used to calculate the percentage contribution of the covariates explaining Jacaranda flowering for each GAMLSS model built. Sensitivity analyses were used to avoid coupling climatic variables with autocorrelation values greater than 0.75 when building models through stepwise model selection. These models were evaluated according to their distribution fit, the number of covariates required to explain flowering observations, the Akaike International Criterion (AIC) and both global and partial deviance. The models with the 15 lowest AIC values were evaluated. The best model chosen was that with the lowest AIC, the lowest deviance, the least number of covariates, and the best fit.

**Table 1:** Distribution fit test for Jacaranda flowering intensity. Lower Akaike Information Criterion values (AIC) and lower degrees of freedom (df) indicate the fit of the dataset to each distribution.

Distribution	df	AIC
Generalized Beta type 1	4	-62.322
Beta	2	-58.385
One-inflated beta	2	-58.385
Beta inflated	4	-54.385
Power Exponential	3	14.345
Box-Cox t	4	56.179
Exponential	1	60.342
Gamma	2	61.747
Normal	2	77.423
Reverse Gumbel	2	77.433
Gumbel	2	79.957
Log normal	2	85.886
Logistic	2	87.45

Wavelet coherence (WaveletComp package) [32] was used to quantify the degree of synchronicity between pairs of climatic variables (rainfall, temperature, and solar exposure) in the time frequency space, and identify processes, scales and constraints potentially influencing Jacaranda flowering in Melbourne, Perth and Sydney. Parramatta and Penrith were excluded for this analysis due to the patchiness of citizen scientist observation effort in the area resulting in too large gaps in the time series. In this analysis, the time domain was represented on the x-axis, whilst the frequency domain was shown on the y-axis, and the colour indicated the degree of coherence (i.e., red outlining high coherence) between these pairs of covariates.

## Results

**Fig 2:**
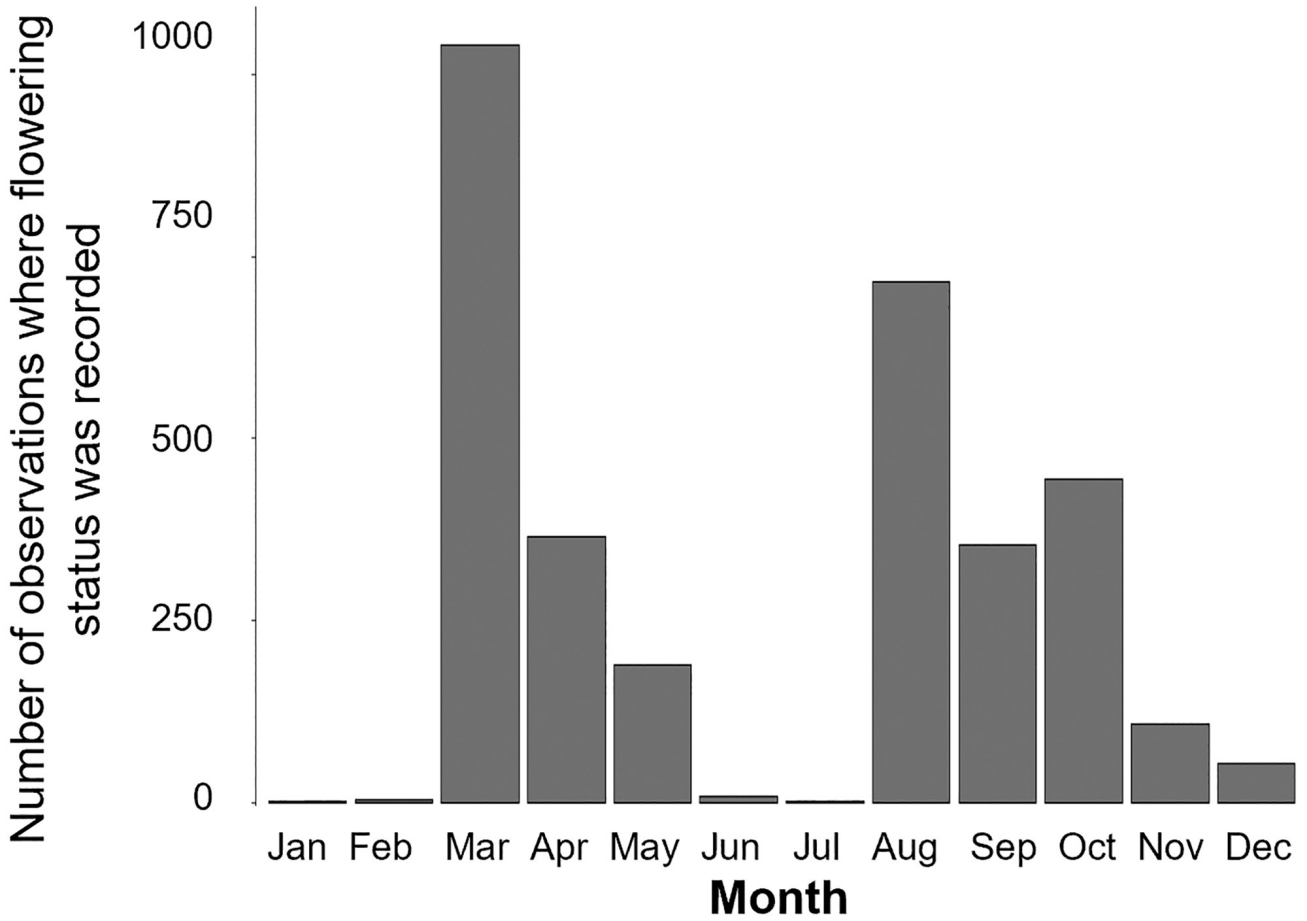
Number of Jacaranda ClimateWatch observations specifying flowering status. Only records in which flowers presence or absence was recorded per month from 2009–2019 are summarised.

The dataset contained recurring months where few or no observations on Jacaranda trees were made (Fig 2). Observations decreased consistently and considerably during these data gaps at the start of the year, during summer (January-February) and in winter (June-July), as a result of decreased citizen scientists’ effort during these months.

### Flowering period and intensity

**Fig 3:**
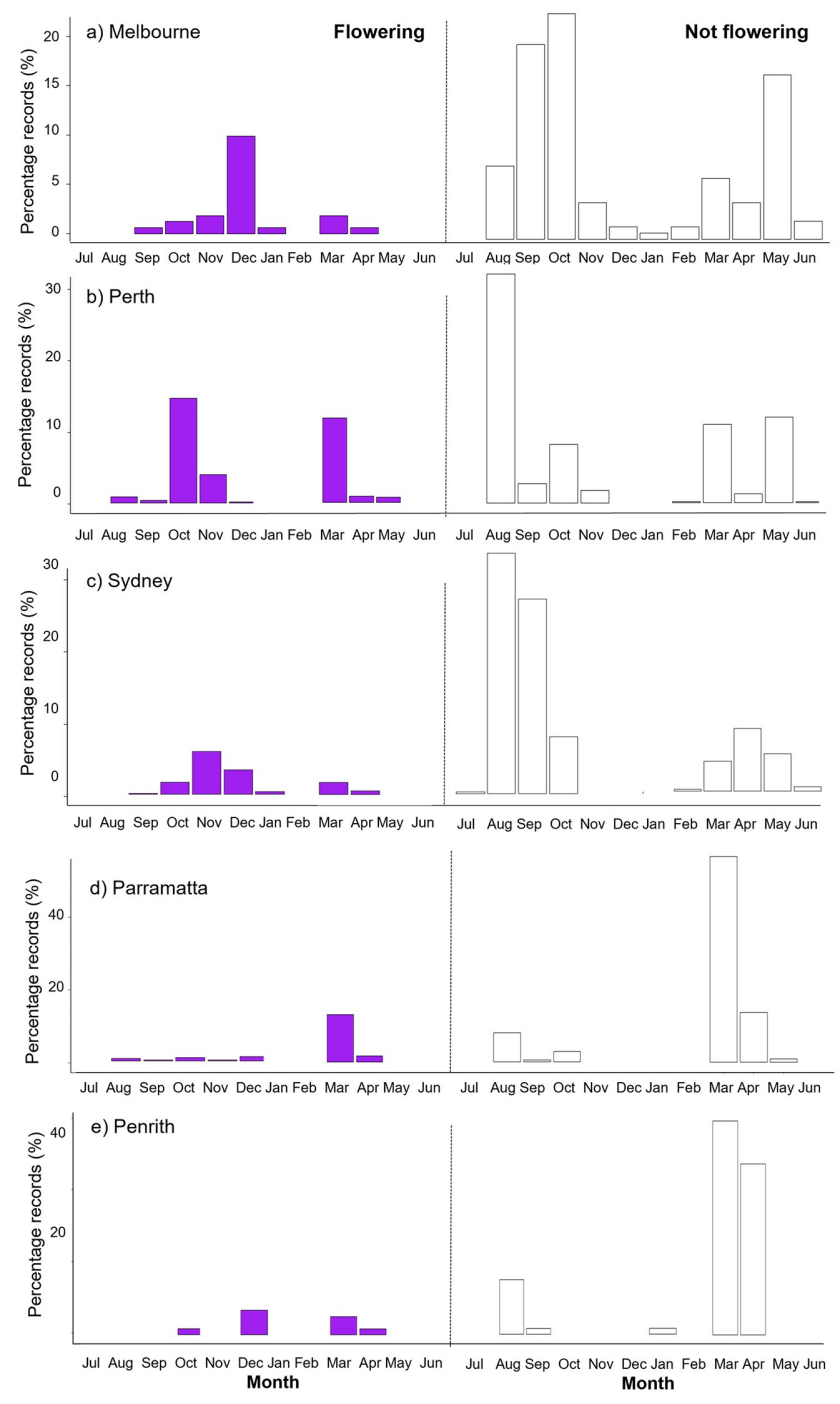
Monthly trends of Jacaranda flowering and non-flowering in the five stations according to ClimateWatch citizen science data between 2009–2019.

Jacaranda flowering period ranged in length and peak-flowering time across stations. In Melbourne, Jacaranda trees were observed flowering between September and April with peak observations made in December. In Perth, Jacaranda trees were observed flowering between August and May with the peak observations made in October. In Sydney, flowering was detected between September and April and the peak flowering was also recorded in October. These observations showed that the flowering season for Jacaranda trees is shortest in Melbourne (~8 months) and longest in Perth (~10 months), and that the peak of flowering occurs sooner after the onset of flowering in Perth and Sydney than in Melbourne (i.e., flowering peaks are reached 2 months in Perth, and 3 months later in Melbourne and Sydney). Due to the patchiness of data collected in Penrith and fewer number of records in Parramatta, flowering period was not determined for these two stations. There were no observations made by citizen scientists in July across Melbourne and Perth, and only a few made in Sydney (Fig 3). Therefore, there could be some flowering patterns not captured by the ClimateWatch dataset.

**Fig 4:**
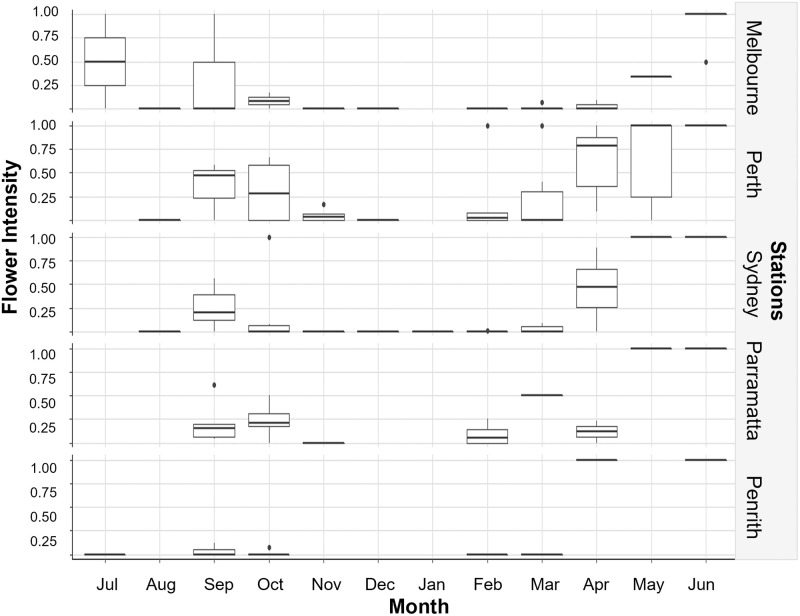
Boxplot outlining the monthly variability in Jacaranda flowering intensity from 2009–2019. A score of 0 indicated that no flowering occurred whilst a score of 1 indicated that flowering was dominant, and flowers were seen in all trees at each station.

Exploring flowering intensity across the years sampled showed that the time, peak and intensity of flowering varied interannually, particularly during the peak time of flowering and towards the end of the flowering season (i.e., larger boxes shown in Fig 4 boxplots indicating larger interannual variability). Perth showed greater flower intensities in peak flowering season in comparison to Sydney and Melbourne (Fig 4).

**Fig 5:**
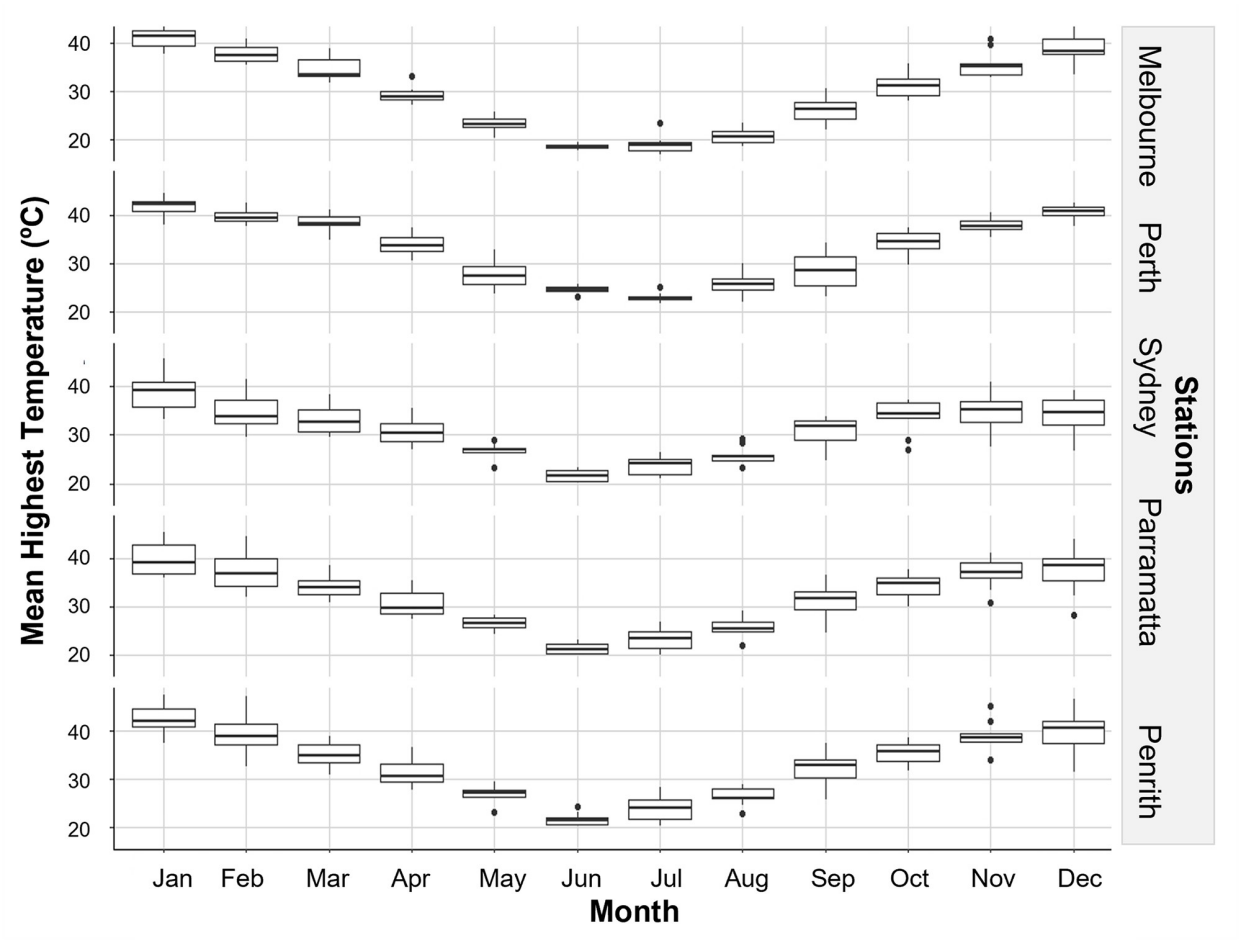
Trend in mean monthly highest temperature from 2009 to 2019 at sampling stations.

**Fig 6:**
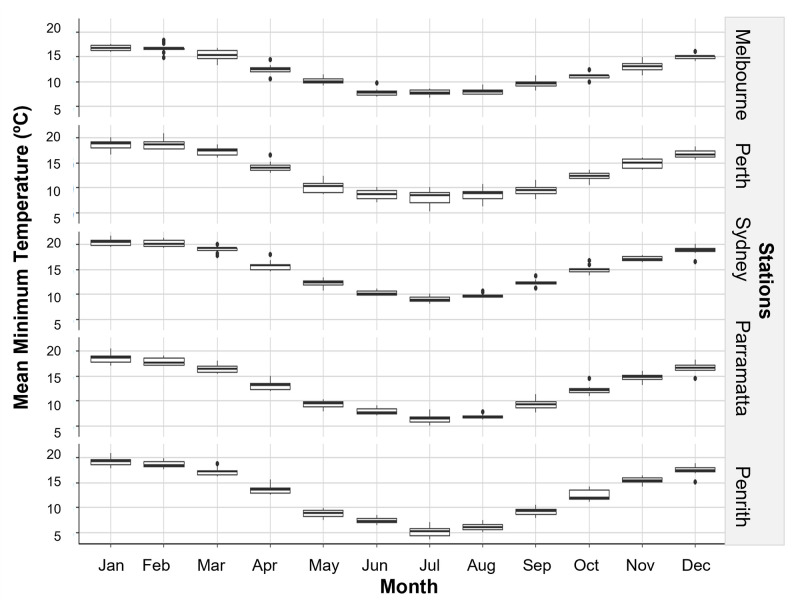
Trend in mean monthly minimum temperature from 2009 to 2019 at sampling stations.

**Fig 7:**
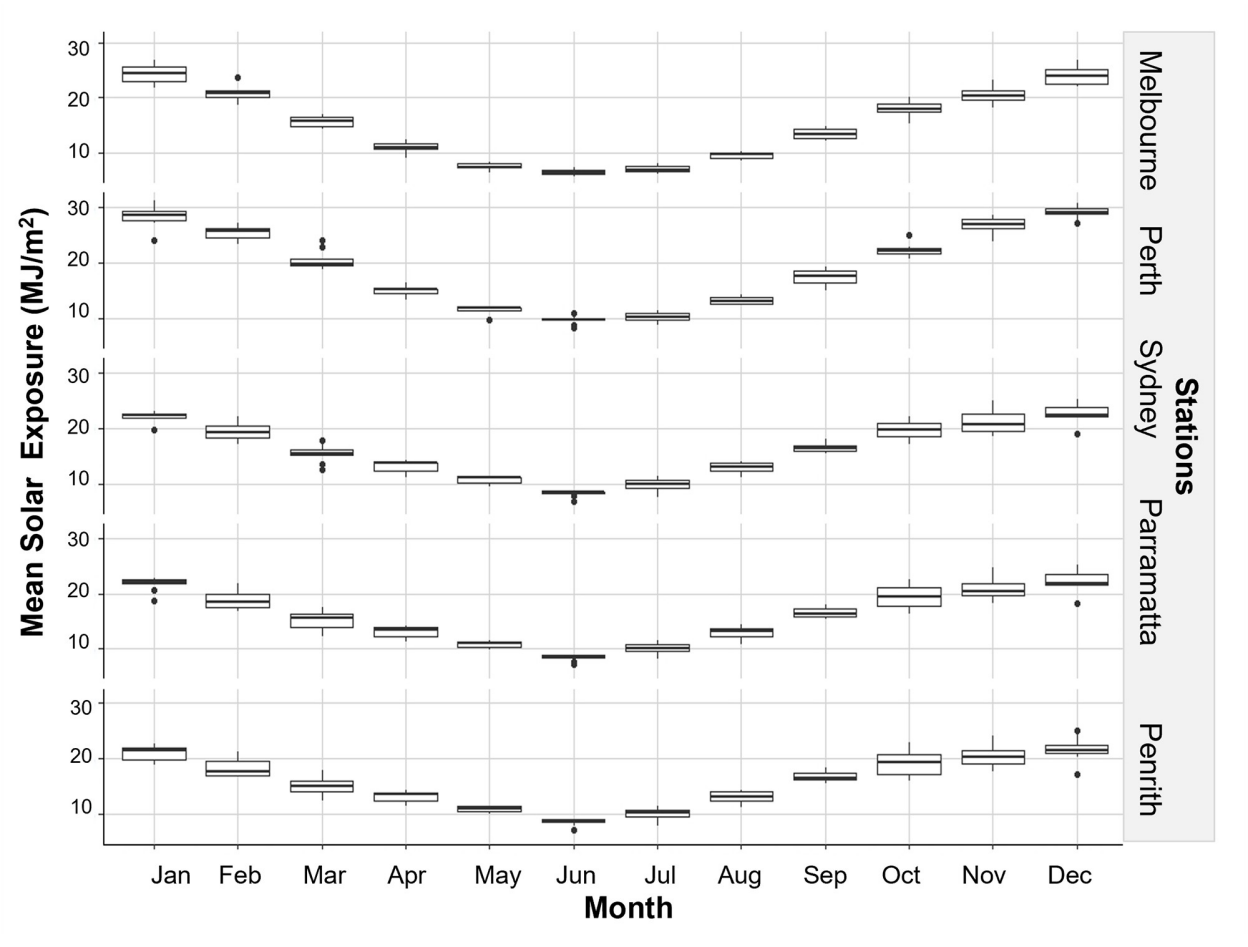
Trend in mean solar exposure between 2009–2019 at sampling stations.

**Fig 8:**
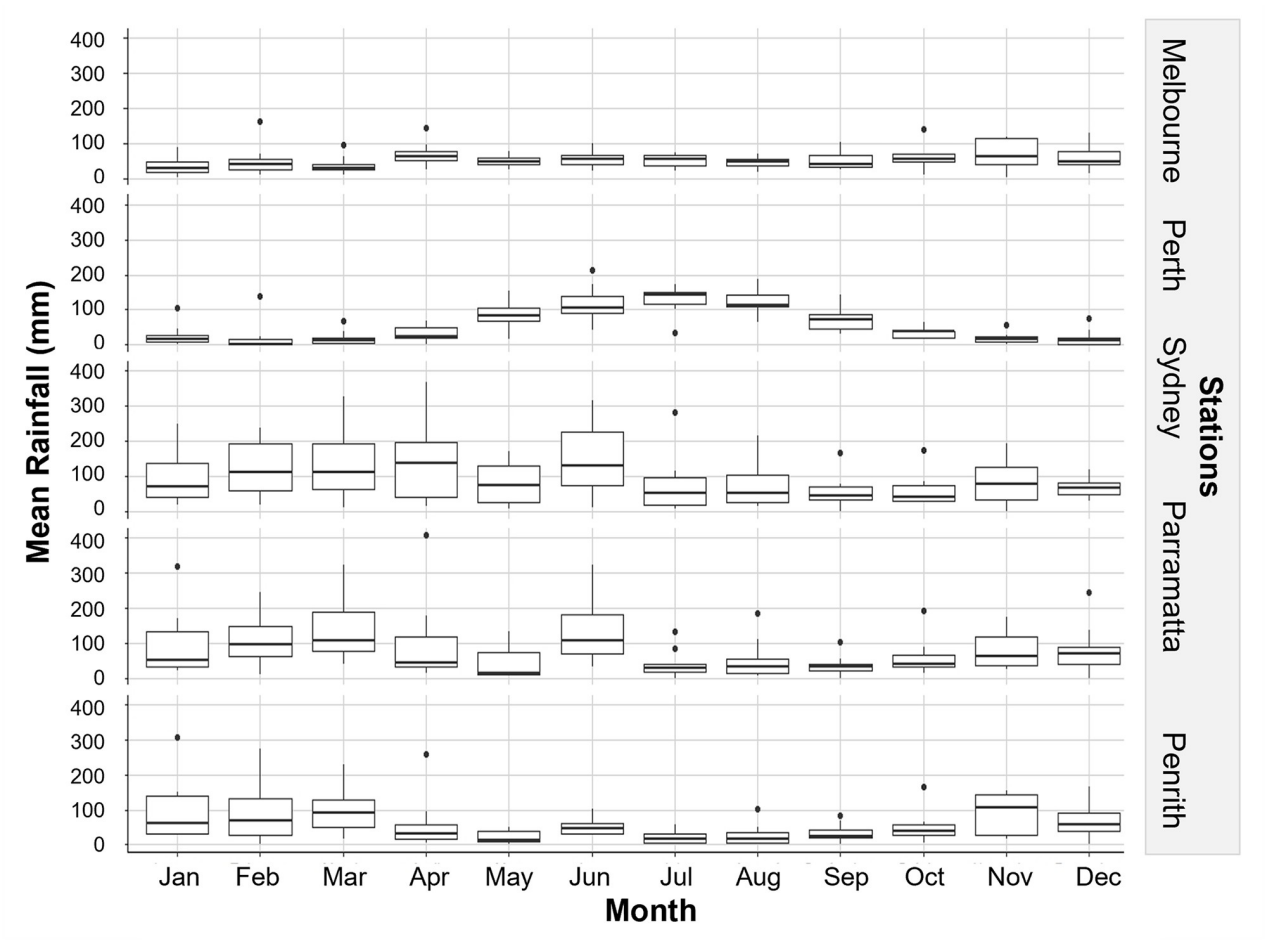
Trend in rainfall between 2009–2019 at sampling stations.

The onset and end of Jacaranda flowering occurred at each of the stations when local maximum temperatures reach and decline 30°C and 25°C respectively (Fig 5) and a few months after the mean minimum temperatures reach and decline below ~15°C (Fig 6). Mean temperatures dipped below 15°C in most stations in May, with the exemption of Sydney where temperature dropped below this threshold in June (Fig 6). Interestingly, the peak flowering intensity coincided with the month where temperatures hovered around 35°C at each station (Fig 5), solar exposure increased and vary the most (>20 MJ/m^2^) (Fig 7), and when conditions became drier after months of heavier rainfall (Fig 8).

### Flowering response to climate

**Fig 9:**
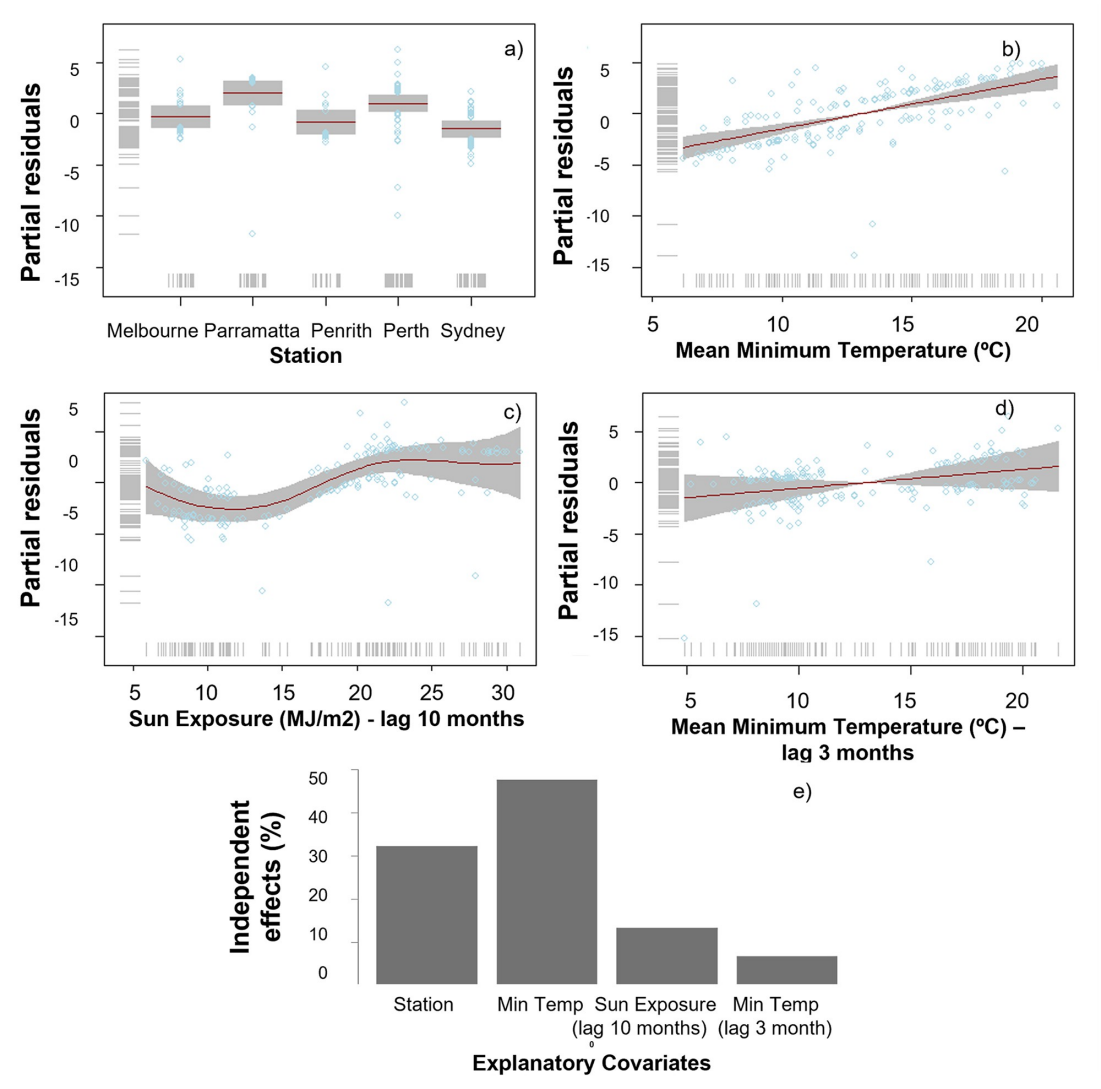
GAMLSS best binomial model explaining the occurrence of Jacaranda flowering between 2009–2019. (a-c) panels showing the partial and the respective percentage contribution of each of the chosen covariates in determining whether or not Jacaranda trees flower (e).

Our models detected clear relationships between climate and Jacaranda flowering onset, period, and intensity, and supported the observations made in the previous section when looking at overlays between flowering and local climate conditions. Flowering occurrence was best explained by differences in station, monthly sun exposure 10 months before flowering, and minimum-mean monthly temperature when flowering commenced and three months prior to flowering (Table 2). Minimum mean temperature and station were the most influential variables (48% and 32% respectively), followed by sun exposure and minimum mean temperature three months prior to flowering (contributing to 20% of the trend in the data) (Fig 9). Flowering was triggered by increasing spring temperatures above 15°C. These models highlight differences in Jacaranda flowering occurrence between Melbourne and Penrith and flowering events occurring in Perth, Sydney, and Parramatta (Fig 9). These linkages are likely a result of local similarities in solar exposure (Fig 7) and temperature ranges (Figs 5 and 6) between 1) Melbourne and Penrith, and 2) Perth, Sydney and Parramatta, and differences across these two groups.

**Table 2:** Binomial models explaining the occurrence of Jacaranda flowering in Melbourne, Perth, Sydney, Parramatta, and Penrith between 2009–2019. The number of asterisks indicate model fit (*Low fit, **Moderate fit, ***Good fit). Bold letters outline the best models with the best fit, the least number of covariates, and the lowest Akaike International Criterion (AIC), Global and Partial deviance (G.Dev and P.Dev). The explanatory variables include Mean Minimum Temperature (MMinTemp), Mean Maximum Temperature (MMaxTemp), Mean Temperature (MTemp), Highest Temperature (HTemp), Rainfall (Rain) and Sun Exposure (SunExp) recorded per month at each weather station. Station is a factor (f) and some variables are smoothed (cs). The number after the variable indicates the lag in months when an association to Jacaranda flowering was detected.

Model	AIC	G.Dev	P.Dev	QQ- Fit
~ f (Station) + MMinTemp + MMaxTemp_2 + cs(SunExp_10)	151.98	129.98	134.14	*
~ f (Station) + Rain + Rain_3 + MMinTemp + cs(SunExp_10)	152.99	128.99	132.21	**
~ **f (Station) + MMinTemp + cs(SunExp_10) + MMinTemp_3**	154.14	132.14	136.41	***
~ f (Station) + cs(SunExp_10) + MMinTemp	154.25	134.25	137.76	***
~ f (Station) + cs(SunExp_10) + MTemp	159.83	139.83	143.01	**
~ f (Station) + cs(MMaxTemp) + cs(MMaxTemp_2) + cs(MMaxTemp_7) + MMaxTemp_8 + MMaxTemp_10	160.70	122.67	128.74	**
~ f (Station) + Rain + MTemp_1 + cs(Rain_3) + cs(HTem_3)	160.83	130.83	134.71	**
~ f (Station) + cs(MMaxTemp_8) + cs(SunExp_10) + MMinTemp_12	161.53	133.52	138.72	**
~ f (Station) + Rain + Rain_1 + Rain_3 + cs(Rain_12) + cs(SunExp_12)	161.64	129.64	132.41	*
~ f(Station) + Rain_3 + MMinTemp + cs(MMaxTemp_2) + cs(SunExp_3)	162.70	132.70	144.35	**
~ f (Station) + MMinTemp + MMaxTemp_2 + cs(SunExp_3)	163.44	141.44	146.05	**
~ f (Station) + MMinTemp + MMinTemp_5 + MMinTemp_7	164.78	144.78	145.89	***

**Fig 10:**
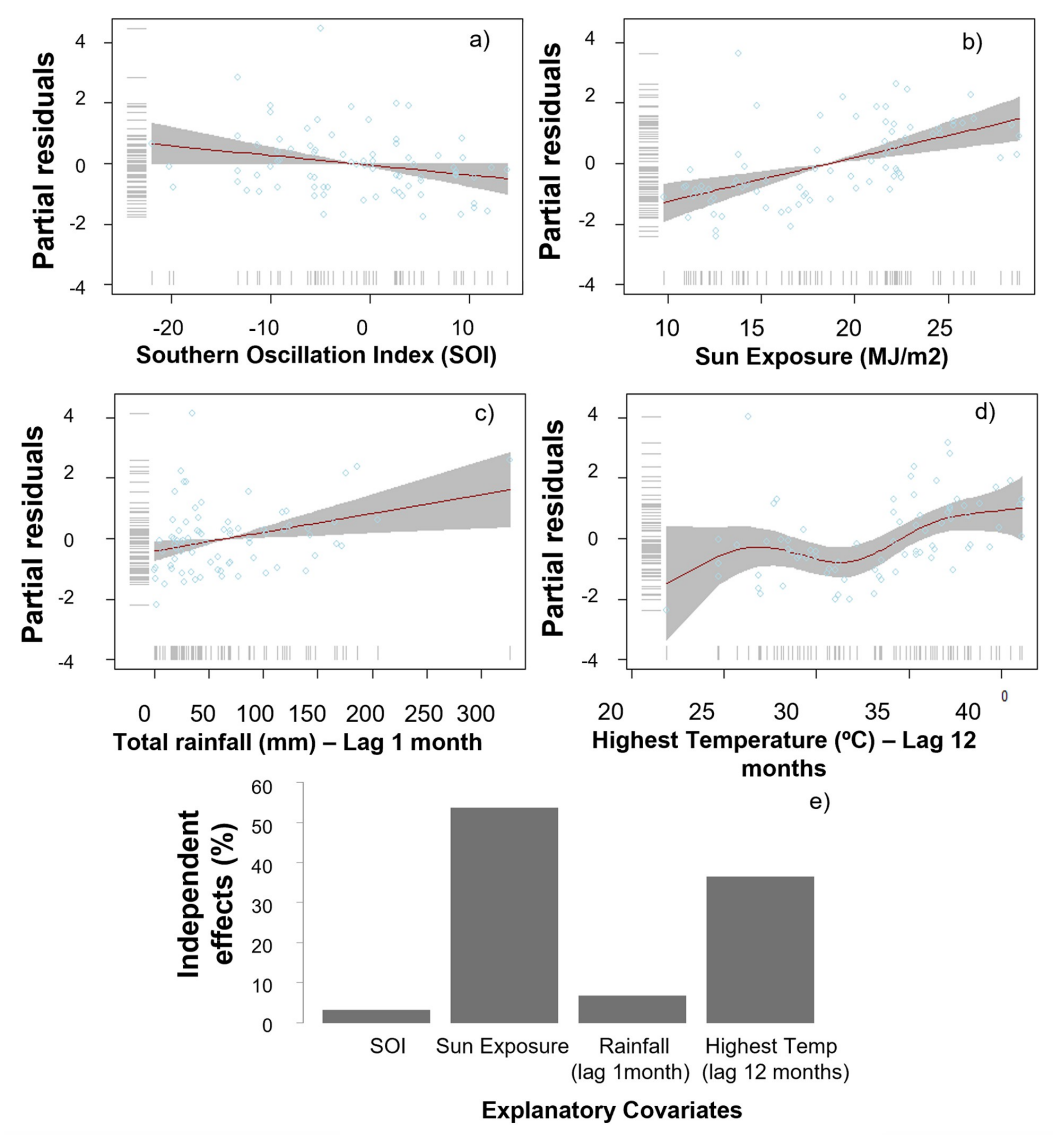
GAMLSS best continuous model outlining the conditions that promoted flowering and triggered maximum flowering intensity (peak flowering events) between 2009–2019. Flowering intensity response per model covariate (a-d) and the respective percentage contribution of each of the chosen covariates in maximising flowering intensity (e). Models were developed using records from Melbourne, Perth, Sydney, Parramatta, and Penrith, and were based on a beta distribution.

Flowering intensity was related primarily to solar exposure and the highest temperature recorded 12 months prior to the observation month, and to a lower degree to increasing rainfall a month earlier and intensity of the Southern Oscillation (SOI) (Fig 10 and Table 3). Contrary to flower onset and period, location was not selected as an important covariate explaining peak flowering intensity (Table 3). Jacaranda maximum flowering intensity occurred at each location when temperatures increased consistently to over 30°C and solar exposure was greater than 20 MJ/m2. Independent effects for solar exposure and temperature were >35%, while rain and SOI only accounted for <10% effect each (Fig 10).

**Table 3:** Continuous models explaining flowering intensity of Jacaranda trees based on a beta distribution in Melbourne, Perth, Sydney, Parramatta and Penrith between 2009–2019. The number of asterisks indicate model fit (*Low fit, **Moderate fit, ***Good fit). Bold letters outline the best models which had the best distribution fit, the least number of covariates, and the lowest Akaike International Criterion (AIC) and both global and Partial deviance (G.Dev and P.Dev, respectively). The explanatory variables include Mean Minimum Temperature (MMinTemp), Mean Maximum Temperature (MMaxTemp), Highest Temperature (HTemp), Mean Temperature (MTemp), Rainfall (Rain) and Sun Exposure (SunExp) recorded per month at each weather station, as well as the monthly Southern Oscillation Index (SOI). The number after the variable refers to number of months prior (lag) the variable was associated to Jacaranda flowering, and cs indicates smooth variables.

*Model*	*AIC*	*G*.*Dev*	*P*.*Dev*	*QQ-Plot*
~ SunExp + cs(Rain_1) + cs(MTemp_2) + Rain_3 + MTemp_3	-289.21	-311.43	-315.21	**
cs(MMinTemp_2) + cs(MMinTemp_3) + MMinTemp_4	-284.88	-306.88	-300.18	**
~ SOI + SOI_12 + cs(SunExp) + cs(Rain_1) + cs(HTemp_12	-282.07	-314.07	-304.94	***
~ cs(Rain) + cs(Rain_1) + HTemp_1 + Rain_2 + cs(HTemp_3)	-280.26	-305.59	-312.26	**
~ MMaxTemp + cs(MMaxTemp_5) + MMaxTemp_6	-277.53	-293.53	-291.***06***	*
~ HTemp_5 + Rain_8 + HTemp_12	-276.33	-283.33	-283.33	**
**~ SOI + SunExp + Rain_1 + cs(HTemp_12)**	-274.91	-292.91	-289.50	***
~ SOI + SOI_6 + SunExp + Rain_1 + Rain_8 + cs(MTemp_5)	-274.71	-296.71	-294.52	***

Interestingly, wavelength coherence analysis showed consistent high coherence (i.e. synchrony) between rain and both temperature and solar exposure at time scales from 9 to 16 months (~256 to ~480 days). These relationships overlapped the time lags at which variables influenced flowering intensity in GAMLSS models. These relationships were mostly out of phase, suggesting a time lag in the temperature and sun exposure to variations in rain.

### Climate trends

Climatic conditions differed across locations (Table 4) with:

- Perth experiencing the warmest temperatures and driest summers, milder temperatures in winter, the shortest period (i.e., 4 months, June to September) of time during which mean monthly temperatures drop under 16°C, high number of days with heavy rainfall (i.e., 31 days per year with rain>10mm), and the strongest and longest solar exposure (i.e., >21 MJ/m^2^ between October and February).
- Melbourne experiencing highly variable weather, harbouring warm summers and cold winters, along the longest period (i.e., 6 months, May to October) of time during which mean monthly temperatures drop under 16°C, consistent rain throughout the year (i.e., highest number of days of annual precipitation, 98 days per year with rain>1mm), and the lowest annual solar exposure (i.e., 181 MJ/m^2^).
- Sydney experiencing generally mild temperatures with five months providing mean temperatures below 16°C (May to September), the highest yearly rainfall and number of days of heavy precipitation (i.e., 1251mm of annual precipitation and 32 days per year with rain>10mm), and moderate solar exposure particularly from November through to February.
- Parramatta experiencing similar temperatures and solar exposures than Sydney (only a couple of degrees cooler), but lower annual rainfall and days of precipitation (i.e., 991mm of annual precipitation and 28 days per year with rain>10mm).
- Penrith experiencing similar temperature and solar exposure trends than Sydney and Parramatta, but registering the coldest winter conditions (4–17°C) of all stations and moderate rainfall.

**Table 4:** Climate patterns in the stations sampled, based on 30 years of data, Australian Bureau of Meteorology [28–30, 33]. The 30-year period used to assess each climate characteristic is outline in parenthesis.

Climate characteristics	Perth	Melbourne	Sydney	Parramatta	Penrith
Mean annual temperature (1961–1990)	19°C	15°C	17.5°C	17.5°C	17°C
Summer temperature range (1961–1990)	17–29°C	14–25°C	18–26°C	17–22°C	16–28°C
Winter mean temperature (1961–1990)	10–18°C	7–14°C	8–17°C	6–18°C	4–17°C
Annual period when mean monthly temperature <16°C (1961–1990)	4 months (June-Sept)	6 months (May-Oct)	5 months (May-Sept)	5 months (May-Sept)	5 months (May-Sept)
Maximum mean monthly solar exposure (1990–2019)	30MJ/m^2^ (Dec)	24MJ/m^2^ (Jan)	23MJ/m^2^ (Dec-Jan)	23 MJ/m^2^ (Dec)	22 MJ/m^2^ (Dec)
Duration of solar exposure >21 MJ/m^2^ (1990–2019)	6 months (Oct-March)	4 months (Nov-Feb)	3 months (Nov-Jan)	3 months (Nov-Jan)	3 months (Nov-Jan)
Mean annual rainfall (1961–1990)	782mm	640mm	1251mm	991mm	873mm
Number of rain days (>1mm) per year (1961–1990)	84 days	98 days	90 days	85 days	80 days
Number of rain days (>10mm) per year (1961–1990)	31 days	18 days	32 days	28 days	25 days

Thus, considering that only temperature ranges in Sydney, Parramatta and Penrith vary slightly, and that sun exposure and rainfall patterns are similar. These three stations are grouped and described together in the discussion and referred to as the Sydney cluster.

## Discussion

Many vegetation phenological studies have focused on the delay or advance of the onset of flowering, and primarily used the time of first flowering to evaluate decoupling of phenological processes [35–37]. However, these dates represent one extreme of the flowering distribution thus are likely susceptible to confounding effects [38]. ClimateWatch citizen science data provided a means to adopt a different approach. Continued monitoring of flowering at large spatial and temporal scales provided a stronger, broader basis, to describe the climatic conditions that drive Jacaranda flowering and identify the most suitable conditions for this species reproduction. Wide ranged citizen science observations at multiple large spatial scales provided a means to identify locations (i.e., cities) where climatic changes may challenge and/or favour the reproductive capacity and dispersal of species, promote dispersal of invasive species, potentially modify the current vegetation cover, and modify the ecosystem service this cover currently provides (i.e., city shading and cooling).

### Flowering responses to climate

*Jacaranda mimosifolia* appears to tolerate a wide range of conditions (mean annual temperatures between 16–24°C) but prefers warm and dry temperate climates where temperatures do not drop below 0°C, colder months do not reach temperatures above 18°C, and average temperatures during warmer months are greater than 10°C [21]. However, little is known about its reproduction and seed dispersal. Experiments and short-term observations [22, 37, 39, 40] suggest that temperature is a key factor influencing germination and flowering in this species with one showing that *Jacaranda mimosifolia* flowering is triggered by prolonged cold exposure (i.e. 15°C for a minimum of three months) [22]. A South African study of first flowering dates between 1927–2019 [23] found that June maximum temperatures (>16.5°C) significantly influenced the timing of flowering commencement.

ClimateWatch is the first long-term study to capture the influence of temperature and the need for cold exposure (15°C) on triggering flowering, as well as the relationship between flowering and the previous year´s climatic conditions (similar to those identified for *Jacaranda copaia*) [22, 41, 42] which likely result from the influence of sun exposure on energy storage, tree development and bud production preceding each flowering season. In years of unfavourable conditions for example, stored reserves may increase (as energy is not used for flowering) and additional energy is available in proceeding years to support increased flowering and/or fruiting [42, 43]. Whilst, during favourable conditions of high solar exposure, increases in metabolisms will influence flowering across seasons [44] as well as plant growth, so that variability in flowering could result from differences in shoot maturity and their respective ability to flower (i.e. in avocado trees for example, autumn and summer shoots produce more flowers than spring shoots [45].

Our models also linked the conditions known to favour Jacaranda to their reproductive cycle and identified that flowering intensity responds to rising temperature and solar exposure, reduced rainfall, and El Niño events (i.e., dry conditions). ClimateWatch findings supported studies linking reproductive success of multiple species of Jacaranda and tropical and temperate trees around the globe to these sets of influencing parameters [23, 42–52], particularly findings by Wright and Calderón [44] and Zhao *et al*. [52] which highlighted the importance of sunlight for Jacaranda flowering.

### The future of Jacaranda throughout Australia

Australia, being a large country with a wide range of climates [53], provides large areas for optimal and tolerable conditions for *Jacaranda mimosifolia* to establish, where this species’ life events (such as flowering) vary temporally and spatially. Such spatial variability in the onset and duration of Jacaranda flowering was detected in our study (e.g., Melbourne peak of flowering was recorded two months after that of Perth and Sydney), and it was likely the result of differences in the timing at which local cues (i.e., temperature and solar radiation) were optimum for flowering at each station sampled. ClimateWatch citizen science observations suggest that currently, Jacaranda trees may be benefiting from the drier conditions and greater exposure to solar energy that occur in Perth, making this city more suitable for flowering than Sydney or Melbourne. However, in the future, considerable changes are expected in each of these cities, and Perth may not provide Jacaranda with the most suitable conditions to flower if the city’s climate transitions from one preferred by this species to one tolerated.

Jacaranda has been planted in Australia both in temperate and tropical climates, particularly in regions close to the coast where the climate is projected to vary considerably [53]. Worldwide, significant range shifts averaging 6.1 km per decade towards the poles and advances of spring events by 2.3 days per decade have been recorded [53]. Overall, temperatures in Australia will continue to rise [54] and with a 2.4°C increase in temperature by 2050 in Australia for example, the extent of temperate climate types would decrease from ~15% to ~9% as parts of the country transition from temperate to arid under a drier future climate limited by water resources (specifically those where climatic boundaries merge, i.e. arid regions boarder dry temperate climates such as is the case of the south-eastern regions of Queensland, (i.e. Fig 3 [53]). This transition and forecast contraction of temperate types in some areas would lead to loss of suitable climate for Jacaranda and other species; and a potential expansion of suitable climate particularly in the southern cooler areas of the country as temperatures rise. For instance, as Melbourne is heading for a 3°C increase by 2070 [54], Jacaranda would be better suited to Melbourne’s future climate than its current cooler and wetter conditions. Therefore, under current trends and range shifts in worldwide climate types [55] suitable conditions for flowering will also shift, threatening the health of trees in cities where species occur at their thermal limits [23]. Differentiating between suitable and tolerable conditions for tree reproductive success is key in identifying cities where tree cover may be lost, and management strategies will be required to overcome the reduction in local ecosystem services (e.g., street tree cover may need to be replaced).

Current trends of rising maximum and mean temperatures decreases in rainfall along the coasts (expect from the northern coast) and cloud cover (i.e. leading to increasing solar exposure) [56], will continue to result in phenological shifts in Australia [5, 14, 35], as has been documented in northern hemisphere [2, 36]. Average temperatures in all stations sampled in this study for example, will increase by approximately 3°C and the number of extreme hot days (>35°C) will double or triple by 2090 [54]. Perth is forecasted to have mean annual temperatures of 21–24°C with 63 days over 35°C, Sydney cluster 18–21°C with 11 days over 35°C and Melbourne 15–18°C with 24 days over 35°C [54]. Changes in rainfall and heat waves will be considerably greater in Perth than in Sydney cluster and Melbourne. Perth will have a reduction in rainfall up to 36% in spring and an increase of extreme heat days (i.e. 20 days per year above 40°C) [54]. These suggest that in the future, Sydney cluster will become more like Perth, Melbourne like Sydney cluster, and Perth will become much hotter. Even though, thermal niches of urban trees can be 38–90% wider than those in which they are found naturally [57] and Jacaranda is known to tolerate a wide range of conditions -including the mean temperatures forecasted for these cities (16–24°C) [21], the increase in extreme events and changes of the lower thermal conditions would likely challenge this species flowering. Since Jacaranda flowering is triggered by cold exposure, these rising temperatures could impact flowering of this species, particularly in cities where mean winter temperatures would start rising above 15°C (i.e., Perth). Thus, changes in climatic conditions are likely to result in changes in flowering Jacaranda, potentially influencing vegetation cover and plant distribution even in urban settings.

### Impacts of phenological changes

Most phenological changes have been recorded around the world during spring [2, 58] altering species interactions, changing community composition and driving major biome shifts [3, 9, 36, 59]. While it is not widely understood how important Jacaranda trees are to other species, large urban trees are recognised as keystone structures that may provide important resources for native wildlife such as birds, particularly those that depend on the availability of food at narrow and/or specific time windows [60]. Some studies have shown the use of Jacaranda for native Australian species. For example, Little Wattlebirds (*Anthochaera chrysoptera*) have been observed in flowering Jacaranda in spring or summer in the east coast of Australia [61], Carnaby’s Cockatoo (*Calyptorhynchus latirostris*) and Baudin’s Black Cockatoo (*Calyptorhynchus baudinii*) have been seen feeding in Jacaranda [62] and higher abundance of nectarivorous birds have been observed in Jacaranda during spring [19]. Honeybees have been found to rely on Jacaranda nectar and/or pollen in Australia as well as other parts of the world [63–65], illustrating a potential role that Jacaranda play in supporting key pollinators.

Following long term studies of climate change impacts observed elsewhere across the globe, it is likely that species that flower during spring in Australia, such as Jacaranda, will change as the climate changes, potentially impacting wildlife that utilise them for food and shelter. As Australia becomes increasingly impacted by threats such as land-clearing and more intense bushfire seasons, Jacaranda and other street trees may become an increasingly valued resource for non-urban species. Moreover, urban trees like Jacaranda support human society by absorbing airborne pollution, supporting natural spaces that promote mental well-being, mitigating climate by providing carbon sequestration [66]. Research shows significant impact to urban phenology [67–69], thus the potential for trees like Jacaranda to reduce surface and air temperature to mitigate the urban heat island effect and climate change warrants further exploration. As such, healthy urbans street trees like Jacaranda provide societal, health and environmental benefits and monitoring their phenology is one way to ensure these benefits are protected. What is more, although, *Jacaranda mimosifolia* is widely planted across the globe [21] in its native range it is considered vulnerable [70] and in parts of South Africa [71] and Queensland, Australia [72], it is considered an invasive species that can potentially out-compete native species [21, 72]. Future work should also explore reproduction and seed dispersal of *J*. *mimosifolia* if we are to improve its conservation status, as well as its invasion risk and apply effective means of control.

### Evaluating the citizen science

Europe has a long history of phenological data collection and networks–many associated with research institutions and government meteorological services [73]—that have often been supported by citizen scientists over several decades [74, 75]. Although the need for a phenological network and the benefit of citizen scientists for phenological observations has long been recognised in Australia [76–78], it was not until the establishment of ClimateWatch in 2009 that this occurred [17]. The program joined other citizen science phenology networks that are filling the void in phenological research across the globe such as the US National Phenology Network, Project Budburst, Zooniverse, Phenoclim, and SeasonWatch [79, 80] to name a few. Thus far, data collection has been complex and patchy throughout the first 10 years of the ClimateWatch program, both due to the variation in multisector partnerships supporting the data collection and the citizen scientists´ time, effort, and ongoing commitment to the program. Overall, the program reflects records captured through personal interest, University coursework and botanic gardens initiatives [81] and includes considerable temporal and spatial gaps as records were collected ad hoc. Regular gaps in flowering and non-flowering ClimateWatch records at the start of the year, during summer (January-February) and in winter (June-July) could be linked to reduced citizen science observation efforts during the Christmas/New Year holiday periods, and reduced guided community activities in cooler periods, respectively. Notable phenology data gaps in the southern hemisphere is detrimental to the discipline by limiting inter-regional comparisons of global climate change impacts [82].

Multiple observers in citizen science initiatives, while increasing capacity to capture data across greater spatial and temporal scales, can lead to considerable irregularities in the level of commitment and frequency of observations [83]. Using additional datasets over the same time-series could help overcome observational noise, however, such datasets do not seem to exist for this species and many of the species monitored through ClimateWatch in Australia. Studies have highlighted that the combination of short observational duration time, as well as infrequent and variable flowering events, have often limited our ability to identify phenological cues [36, 44], yet a decade of citizen science observations used in this study, provided the first insight into multiregional climatic cues that influence and determine flowering of Jacaranda in Australia. This highlights how useful ‘people power’ in citizen science data collection can be for increasing scientific knowledge.

Several aspects of the program could be addressed for improvement and increased potential to contribute towards climate change research for other indicator species. The major limitation being lack of long-term funding and resources (i.e. a full-time long-term team). ClimateWatch was run with one program manager at different times throughout its history and was supported by intermittent funding from government agencies, private trusts, and corporations. Having few dedicated staff and lack of long-term funding are common attributes for citizen science projects run by not-for-profit organisations in Australia and elsewhere [84, 85]. The gaps in the national ClimateWatch dataset could significantly be addressed by additional resources to support ClimateWatch coordinators in each state and territory to undertake local, ongoing ClimateWatch monitoring and engagement with wider participants of the community, such as environmental groups, outdoor educational operators, national parks staff, catchment management authorities, local government bodies and private landholders. Enhancements to technology could also support resource-intensive data validation processes and improve communications with ClimateWatch users, providing reminders and messages of encouragement to continue regular observations.

Citizen science has a long history in Australia when it comes to the collection of climate data. Without the Bureau of Meteorology’s network of volunteer weather observers that have been collecting climate readings for over 100 years [86], Australia’s capacity to forecast and respond to climate events, including flooding and heat extremes, would be very limited. To respond to phenological impacts effectively across Australia, a similar large-scale, government supported network of volunteers would benefit national capacity to act on climate change impacts. What is more, citizen science programs like ClimateWatch, could enhance national efforts by supplementing government records with those made by the Australian public through smartphone app and web technologies. Monitoring the phenology of other species groups where data is lacking, such as mammals and invertebrates [5] would help document changes in taxonomic groups where phenological shifts are poorly understood. As discussed by Chambers *et al*. [14], although our dataset is not yet long or spatially broad enough to draw reliable conclusions or parameterize predictive models, it serves as a strong baseline. With adequate resources, citizen science programs such as ClimateWatch could help facilitate some of this much needed data capture across Australia.

## Conclusion

Our models provide the first attempt to describe the climate drivers for *Jacaranda mimosifolia* flowering in the southern hemisphere using citizen science collected data and highlight the role of sun exposure and temperature as climatic drivers that determine reproductive success of this species, and therefore its ability to provide cities with key ecosystem services including alternative food sources for birds and honeybees. Based on climatic predictions where temperatures will rise, cloud cover will increase and rainfall will decrease, plants like Jacaranda that require cold exposure to flower, will be impacted, particularly in cities like Perth where such changes will be challenging for this species’ thermal requirements. Jacaranda may benefit in areas like Melbourne, where lower thermal limits will still be provided, and warmer summers may aid tree growth and development. These findings were supported by both experimental and short-term studies conducted in other countries.

Even though the ClimateWatch citizen science program is in its early stages, as Australia’s first attempt at a national phenology network, it is producing useful understanding of phenology and can continue to do so if quality cross-sectoral collaborations and long-term funding are secured. ClimateWatch citizen scientists have helped address the difficulty of obtaining scientific data at scales that individual scientists cannot attain and substantiate that expert opinion is effective in the selection of indicator species for citizen science phenological monitoring across the country. The efforts of ClimateWatch citizen scientists monitoring Jacaranda, as well as the other 150+ species on the ClimateWatch program, is valuable, and collecting a further ten years of data across all months of the year will only increase the integrity of the Jacaranda dataset further. Long-term collaborations among scientists, the public, government agencies and organisations are required if scientific understanding of the effects of climate change on species’ phenology are to be realised across Australia.
